# Investigation of work of adhesion of biological cell (human hepatocellular carcinoma) by *AFM* nanoindentation

**DOI:** 10.1007/s12213-016-0089-8

**Published:** 2016-05-07

**Authors:** Xinyao Zhu, Nan Zhang, Zuobin Wang, X. Liu

**Affiliations:** 1 School of Engineering, University of Warwick, Coventry, CV4 7AL UK; 2 International Research Centre for Nano Handling and Manufacturing, Changchun University of Science and Technology, 7089 Weixing Road, Changchun, 130022 People’s Republic of China

**Keywords:** JKR, Young’s modulus, Work of adhesion, Atomic force microscope, Fullerenol

## Abstract

In this study, we presented an investigation of mechanical properties by AFM nanoindentation on human hepatocellular carcinoma cells treated with fullerenol for 24, 48 and 72 h. AFM nanoindentation was routinely applied to investigate the morphology and biomechanical properties of living carcinoma cells, and adhesion phenomena (negative force) were detected in the obtained force-displacement curves. Conventionally, Hertz contact model has been widely used for determination of cell elasticity, however this contact model cannot account for adhesion. Alternatively, JKR contact model, as expected for adhesion circumstance, has been applied to fit the obtained force-displacement curves. In this investigation, we have derived both the work of adhesion and the elastic modulus of biological cells (human hepatocellular carcinoma) under fullerenol treatment. The results show that the chosen JKR model can provide better fitting results than Hertz contact model. The results show that both Young’s modulus and work of adhesion exhibit significant variation as the treatment time increases. The calculated mechanical properties of elastic modulus and work of adhesion can be used as an effective bio-index to evaluate the effects of fullerenol or other anticancer agents on cancer cells and thus to provide insight into cancer progression in the treatment.

## Introduction

Fullerene family has been playing an important role for potential applications in biomedicine such as cancer diagnosis and therapy [1–3]. The fullerenol can induce apoptosis process which is associated with cytoskeleton disruption [4]. Cancer cell affected by fullerenols could exhibit variations in mechanical properties such as elastic stiffness and these changes in cancer progression are helpful to understand the individual differences between normal and cancer cells [5, 6]. The atomic force microscope (*AFM*) nanoindentation can offer an accurate mechanical measurement of individual living cells [7–9]. The adhesion phenomenon, characterized as negative force in the experimental force-displacement curves obtained in *AFM* nanoindentation, was widely reported over the last two decades [10–13]. The adhesion behavior of cells with other nanoparticles is crucial for the biocompatibility of implants [14]. In recent years, it becomes clear that adhesion molecules are involved in tethering cells to specific locations [15]. Adhesion molecules are transmembrane molecules that are linked to cytoskeletal elements (actin) [16]. Since fullerenols have appreciable effect on cytoskeletal structures, the adhesion property of cancer cyto-membrane may also alter due to fullerenol treatment.

Hertz contact model has been routinely used for the determination of cell elasticity based on *AFM* nanoindentation. Since Hertz model assumes that there is no adhesion existing in interfacial area, the elasticity analysis based on Hertz model could not account for the tip-cell adhesion. Pioneering studies of adhesive contact between compliant spherical bodies (or rigid sphere and compliant body) have been developed by Johnson [17].

In this study, human hepatocellular carcinoma cells (SMCC-7721) treated with fullerenol [C_60_(OH)_24_] under different time period (24, 48, 72 h) are presented. *AFM* nanoindentation is utilized to obtain the force-displacement (*F-d*) curve. *JKR* model was applied to fit the retraction part of *F-d* curves and the corresponding Young’s modulus and work of adhesion were obtained. We found that adhesion phenomenon is dependent on time duration of fullerenol treatment. The control cell and the cells exposed to fullerenol for 24 h showed insignificant adhesion while the rest two kinds of cells exhibited conspicuous adhesion. The fitted *JKR* model provides good agreement with the experimental results. The changes of the determined work of adhesion (*Δγ*) due to different periods of fullerenol treatment are provided.

## Methodology

### Cell Preparation

SMCC-7721 cells were obtained from Roswell Park Memorial Institute (RPMI)-1640 media with 10% of fetal bovine serum (FBS) and antibiotics (penicillin–streptomycin solution). The commercial water-soluble fullerenol powder with the general formula C_60_(OH)_24_ was dissolved in deionized water at a concentration of 2.7 μM/ml, and it was then diluted with RPMI-1640 media with 10% of FBS to 0.53 μM/ml, which was used for the fullerenol treatment solution stored at 41°C. The Maintenance of SMCC-7721 cells and sample preparation have been described in details elsewhere [6]. In this study, we labeled control cells as cells *A*, which were not exposed to fullerenol and being cultured for 24 h in the physiological solution, and marked cells exposed to fullerenol for 24, 48 and 72 h as cells *B*, *C* and *D* respectively. Cells A consist of 7 cells, cells B (treated for 24 h) are 12 cells, and cells C (treated for 48 h) and D (treated for 72 h) are 7 cells each.

### Atomic Force Miscroscopy

The module of the AFM employed in this study is JPK NanoWizards 3 BioScience mounted on an inverted optical microscope, allowing the AFM and optical microscope imaging simultaneously. The criterion for cantilever selection is that the compliance of the cantilever should be around the range of the sample compliance. For very soft and delicate cells, the softest cantilevers are available with spring constants ranging from 0.01 to 0.03 *N*/*m* (JPK Application Note). Therefore, a silicon nitride cantilever whose spring constants is 0.03 *N*/*m*, was chosen for cell-tip indentation in this paper. The probe is a square pyramid tip with a half-opening angle of 25° (half-angle to face), and its radius and height are 10 *nm* and 2.5–8 μm respectively. During the indentation, the loading and retracting speeds were kept constant at about 2.5 μm/*s* for all experiments to avoid viscosity effect.

## Theoretical Model

**Fig. 1:**
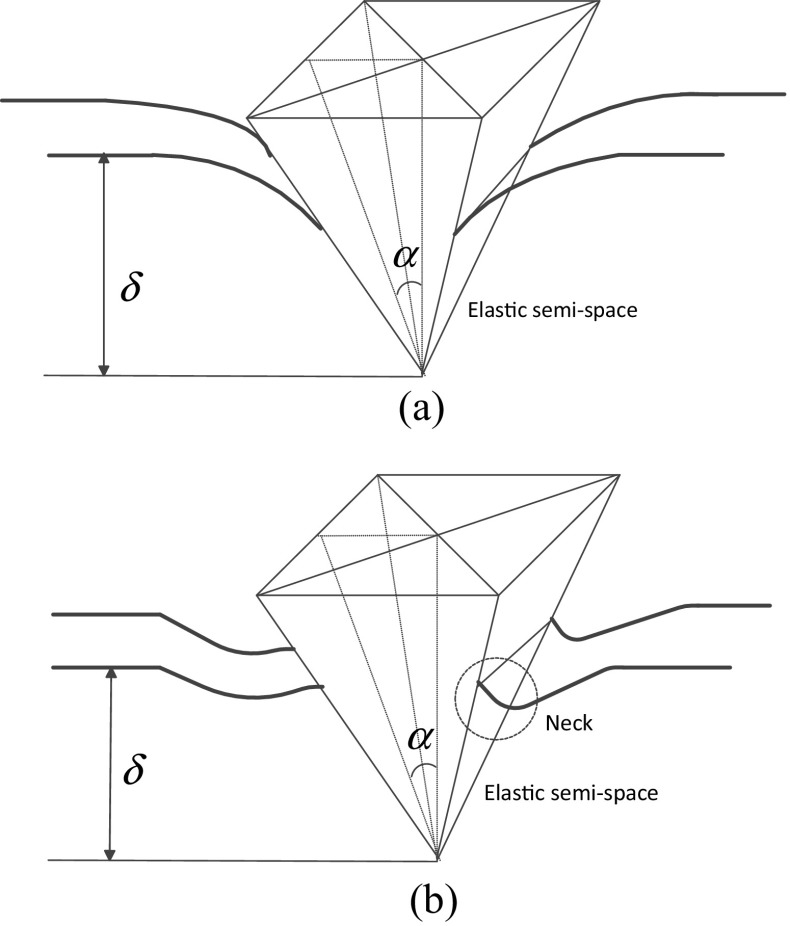
Scheme of (**a**) non-adhesive and (**b**) adhesive contact between a square pyramid and a compliant semi-infinite space. *α* denotes the half-angle to face. The neck area is ascribed to adhesion force

Figure 1 illustrates the scheme of a soft semi-space material indented by a square pyramid tip. During the approach and retraction processes, the viscosity effect can be neglected, and only elastic deformation is considered, as long as the indentation is performed in a time loner than the force relaxation time of the cell-AFM tip system [10]. Compared to the size of the *AFM* tip, the cell could be treated as a semi-infinite space. For a non-adhesive contact, Sneddon [18] gave a relationship between the force *F* and indentation depth *δ* as

1 $$ F=\frac{4 Etan\alpha}{\pi^{3/2}\left(1-{v}^2\right)}{\delta}^2 $$

where *E* and *ν* denote the Young’s modulus and Poisson’s ratio respectively. For biomaterials, we always treat it as incompressible and hence *ν* = 0.5. If the adhesion between the tip surface and cyto-membrane is taken into consideration as shown in Fig. 1(*b*), the counterpart relationship is as [19]

2 $$ F=\frac{4 Etan\alpha}{\pi^{3/2}\left(1-{v}^2\right)}{\delta}^2-\frac{32\Delta \upgamma \tan \alpha }{\pi^2 cos\alpha} $$

where *Δγ* denotes work of adhesion which means the energy needed (or released) when unit area of interface is created (or merged). Owning to the second term in Eq. (2), negative value of force is manifested when the indentation depth is small, which is commonly observed in many *AFM* nanoindentation experiments on living cells [10, 11]. As suggested, when *Δγ* equals zero (no adhesion), Eq. (2) will reduce to its non-adhesive counterpart, i.e. Eq. (1).

## Results and Discussions

### Analysis of the F-d curves

**Fig. 2:**
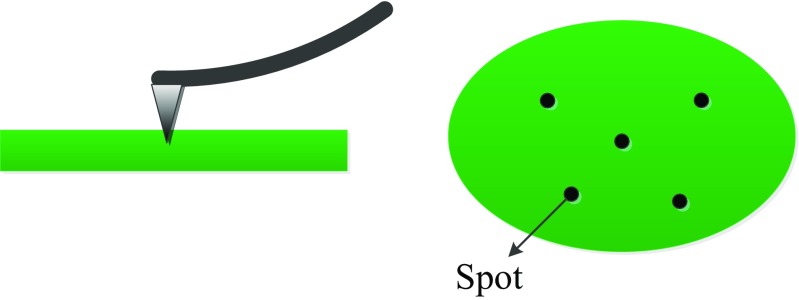
Illustration of AFM probe indenting a cell

**Fig. 3:**
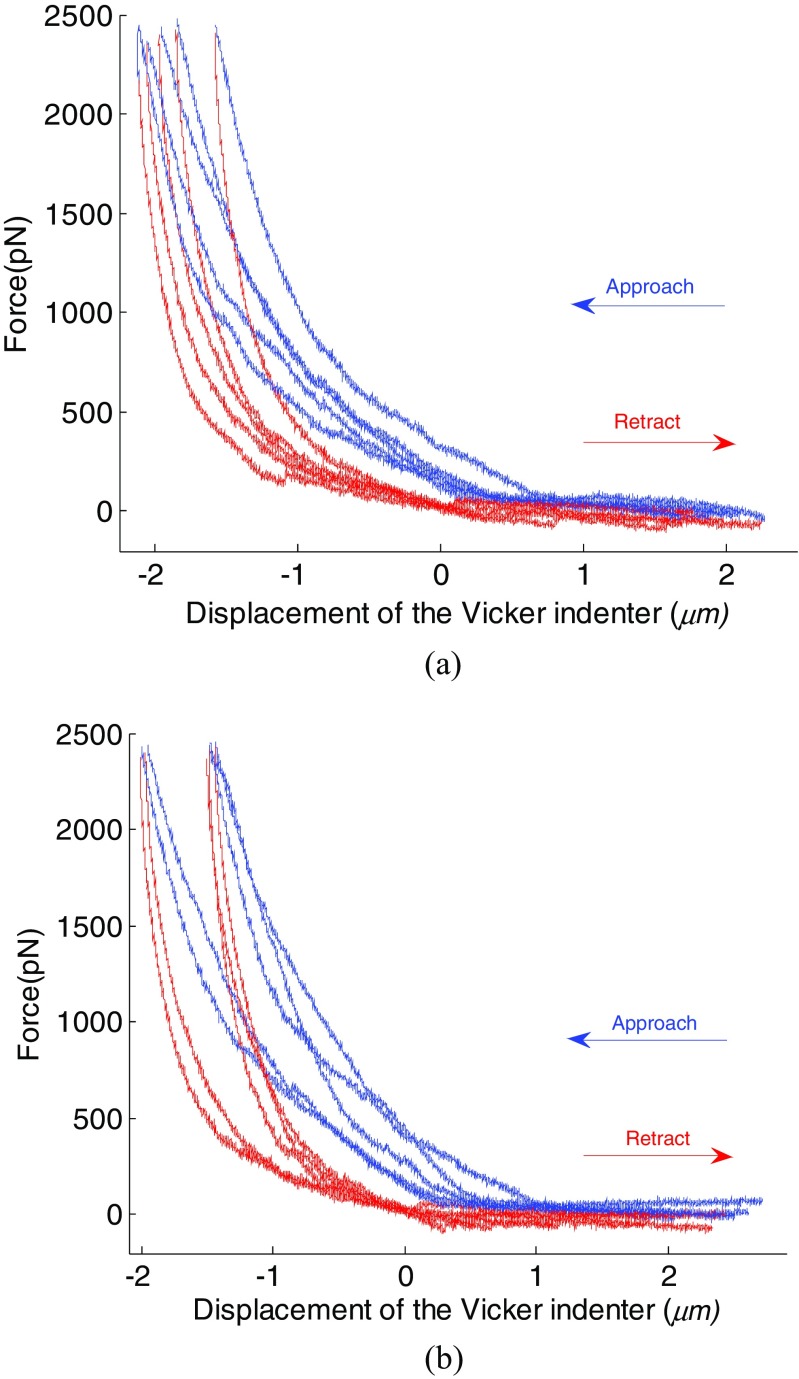
Typical F-d curves corresponding to (**a**) repeated indentations at the same spot and (**b**) different indentation positions within the same cell

During nanoindentation measurements, one live cell was generally indented 3–5 times at the same spot and this was repeated at 5 different spots as illustrated in Fig. 2. The typical force-deformation curves (*F-d*) by the *AFM* nanoindentation are shown in Fig. 3. It can be seen, from Fig. 3a, that indenting on the same spot of the cell, the obtained *F-d* curves vary insignificantly. While indenting on slightly different spots of the same cell, the corresponding *F-d* curves differ only by a small margin with each other as shown by Fig. 3b, indicating that the location of the indentation spot may not introduce much variation of the concerned measurements. The curve shift either from the same spot or different spots is generally less than 1μm and is likely attributed to different indentation positions which correspond to different cell height. Due to the soft nature of living cells, this curve shift is much expected. The calculated Young’s modulus, as shown in Table 1, has a similar standard deviation for both on the same spot and at different spots from the same cell. Therefore, the results presented here are dependent only on the types of cells not the location of indentation spots.

**Table 1:** Young’s modulus from cell *A2* (Unit: *kPa*)

Time \ spot	1st	2nd	3rd	4th	Avg (Std)
1	2.697	2.403	2.171	2.096	2.34 (±0.27)
2	2.033	2.113	2.253	2.179	2.14 (±0.09)
3	2.146	2.82	2.565	2.389	2.48 (±0.28)
4	2.401	2.024	2.321	2.512	2.31 (±0.21)
5	2.568	2.356	2.86	2.028	2.45 (±0.35)

**Fig. 4:**
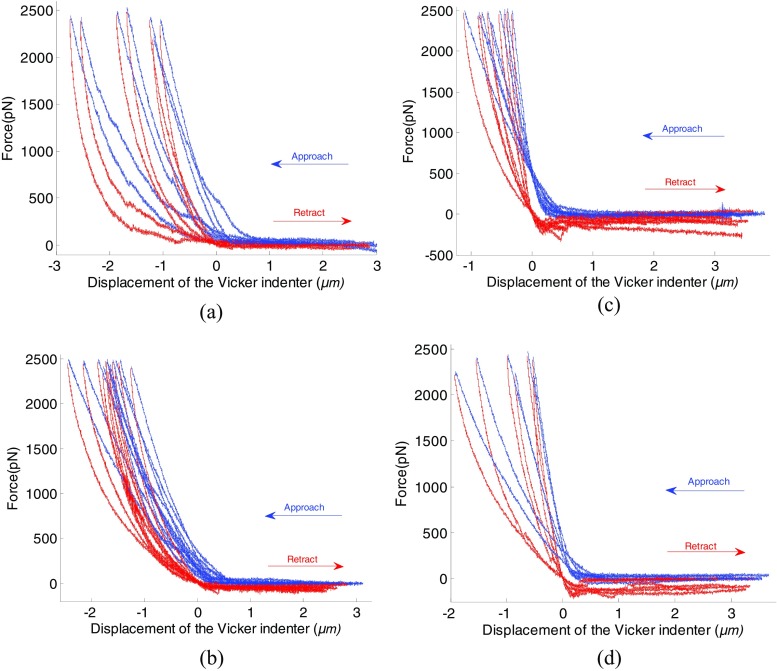
Force-displacement curves obtained by *AFM* nanoindentation on (**a**) control cells (7 cells), cells exposed to fullerenol for (**b**) 24 h (12 cells), (**c**) 48 h (7 cells) and (**d**) 72h (7 cells) respectively

Figure 4 presents result of force-displacement curves for the four types of SMCC-7721cells after the above mentioned treatments.

The shift in displacement is very likely ascribed to different heights of different cells. It should be borne in mind that Fig. 4a-d correspond to four different cells respectively (i.e. control cell, 24, 48 72 h cells). Taking Fig. 4a as example, the seven cells correspond to seven control cells, and they are very likely to differ in cell height, which mean the contact point would differ in each *F-d* curve.

The maximum indentation force was set of approximately 2500 *pN* regardless of type of the cells while the maximum indentation depth varies from cell to cell, and the maximum indentation depth was in a range of 1 to 2 μm. For the cell *C* and *D*, adhesion force is characterized by the negative force region during retraction of *AFM* indenter as illustrated by the red line in Fig. 4c and d. However, adhesion force is not noticeable for cells *A* and *B* as shown in Fig. 4a and b from the retraction.

### Control cells and cells exposed to fullerenol for 24 h (Non-adhesion case)

Since adhesion phenomenon is insignificant in cells *A* and *B*, the non-adhesive Hertz contact model (Eq. (1)) is adopted to fit the retraction part of the force-displacement curve corresponding to cells *A* and *B*.

**Fig. 5:**
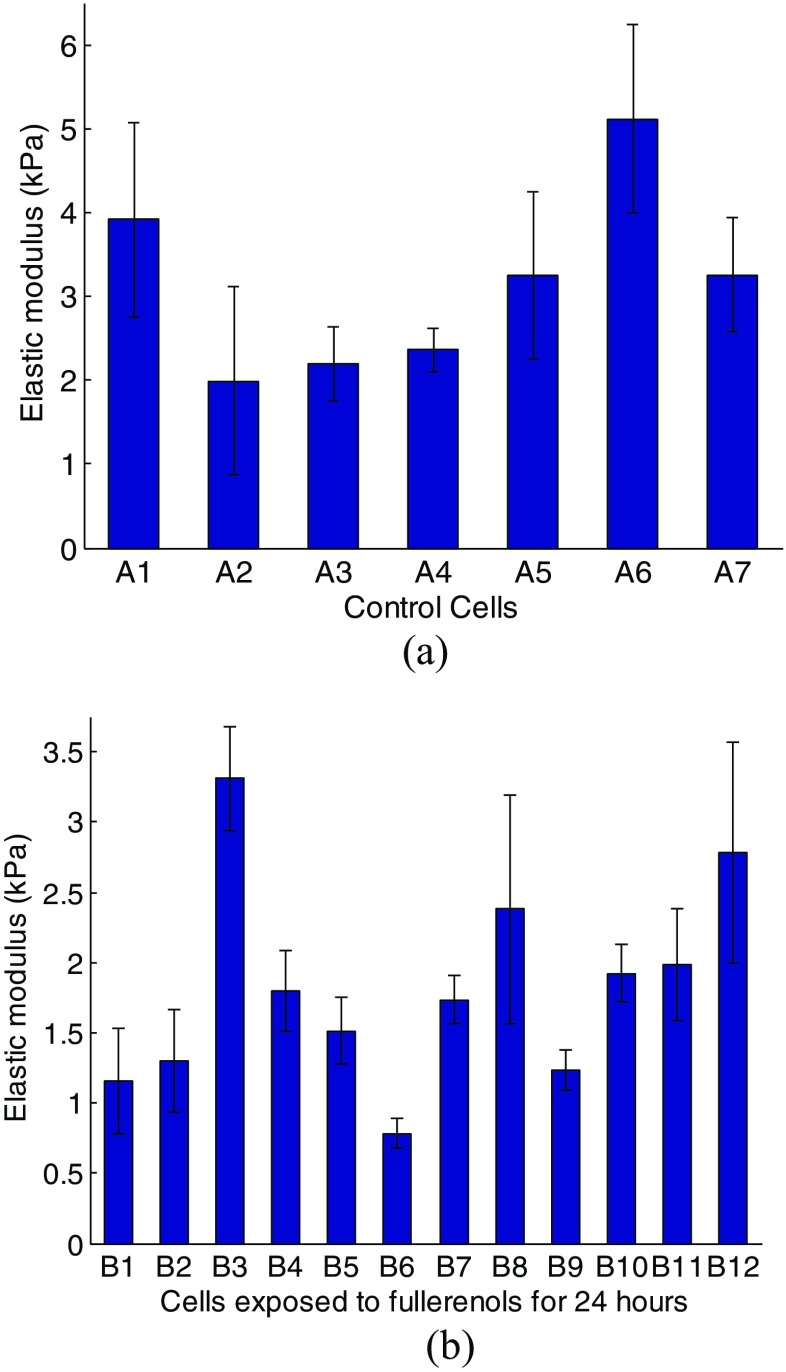
The determined Young’s modulus for (**a**) control cells and (**b**) cells exposed to fullerenol for 24 h. The data are presented as average values with standard deviations

**Fig. 6:**
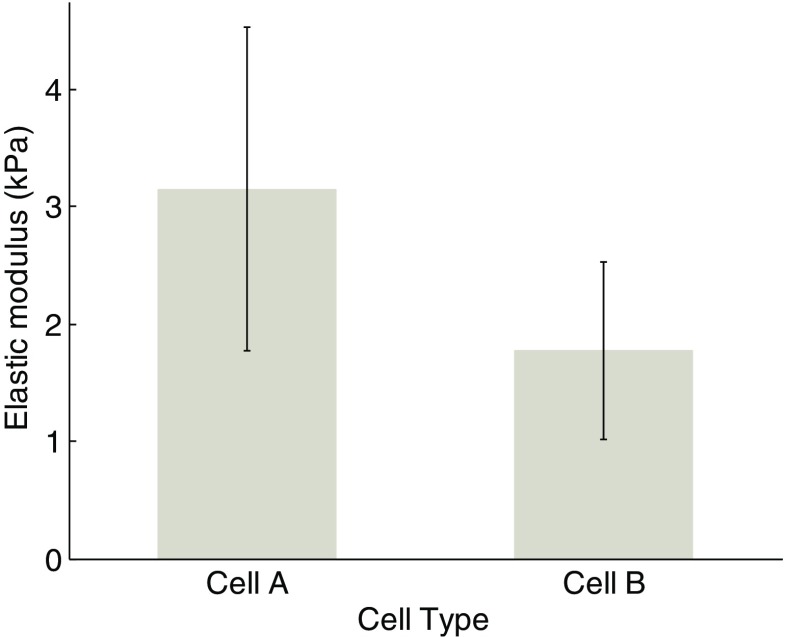
The comparison of determined Young’s modulus between cell **a** and **b**

Tables 1 and 2 give the extracted Young’s modulus at five different positions within the same cell. Four indentations were repeated in every position. It can be seen that the determined Young’s modulus values from different positions within the same cell remain fairly steady. The calculated Young’s modulus values for all cells are shown in Fig. 5. Each individual bar represents one cell, expressed as average ± standard deviation. It is noted that the determined Young’s modulus varies significantly from one cell to another. For cell *A*, the Young’s modulus mainly ranges from 2 to 3.1 *kPa*, and only Young’s modulus values of cell *A*1 and *A*6 are beyond this range by a considerable margin. For cell *B*, most Young’s modulus ranges from 1 to 2 *kPa* or even lower, and only Young’s modulus values of *B*3, *B8* and *B12* are beyond this range. Figure 6 shows the overall comparison result for the control cells and the cells treated for 24 h. The data indicate that fullerenol decreased the elastic modulus by 43% after 24 h treatment, suggesting that cells treated with fullerenol become considerably compliant.

**Table 2:** Young’s modulus from cell *B1* (Unit: *kPa*)

Time\ spot	1st	2nd	3rd	4th	Avg (Std)
1	1.76	1.811	1.879	1.109	1.64 (±0.36)
2	1.332	1.562	1.663	1.531	1.5 (±0.14)
3	1.699	1.773	1.715	1.82	1.75 (±0.06)
4	1.831	1.923	2.234	2.324	2.08 (±0.24)
5	1.985	2.099	2.065	1.887	2.01 (±0.09)

### Cells exposed to fullerenol for 48 and 72 h (Adhesion case)

**Fig. 7:**
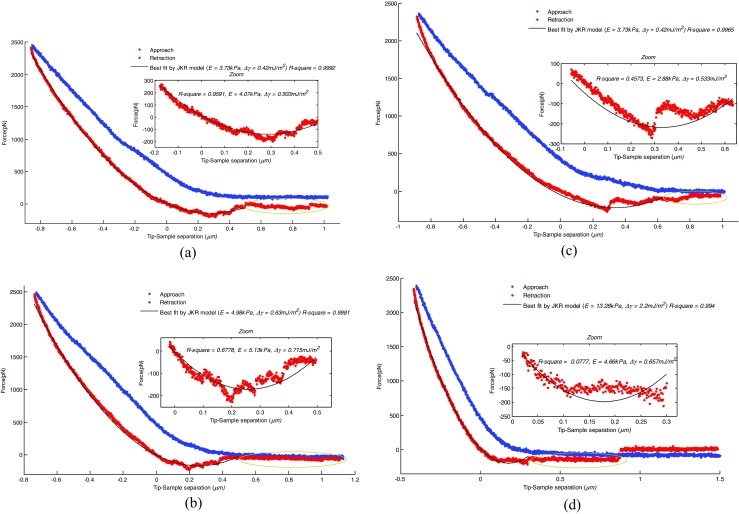
Typical force-displacement curves and the best fitting curves by using *JKR* model. The zoom box denotes the “local fitting”

Figure 7 presents typical results of the force-displacement curves obtained by *AFM* nanoindentation on cells *C* and *D*, and the best fitting curves by using *JKR* model for the retraction parts. Sudden jumps of indentation force occur during retraction process, which is characterized by the “wave” as shown in the zoom box area. These sudden variations of the force can be ascribed to discontinuous decrease of the contact area between the tip and cell membrane. It is due to one tethering of cell membrane to *AFM* tip surface followed by a sudden detachment and tethering to another contact line [19]. Since fitting of *F-d* curve with discontinuous steps will cause error in the estimation of work of adhesion, the last section of *F-d* curve corresponding to considerable discontinuous adhesive force, as indicated by oval circle in Fig. 7, were discarded from fitting [19, 20]. Moreover, if the curve itself consists of significant and abrupt force discontinuity, it will be discarded for numeral statistics too. In general, the *JKR* model can best describe the experimental results of the unloading curve as shown above.

**Fig. 8:**
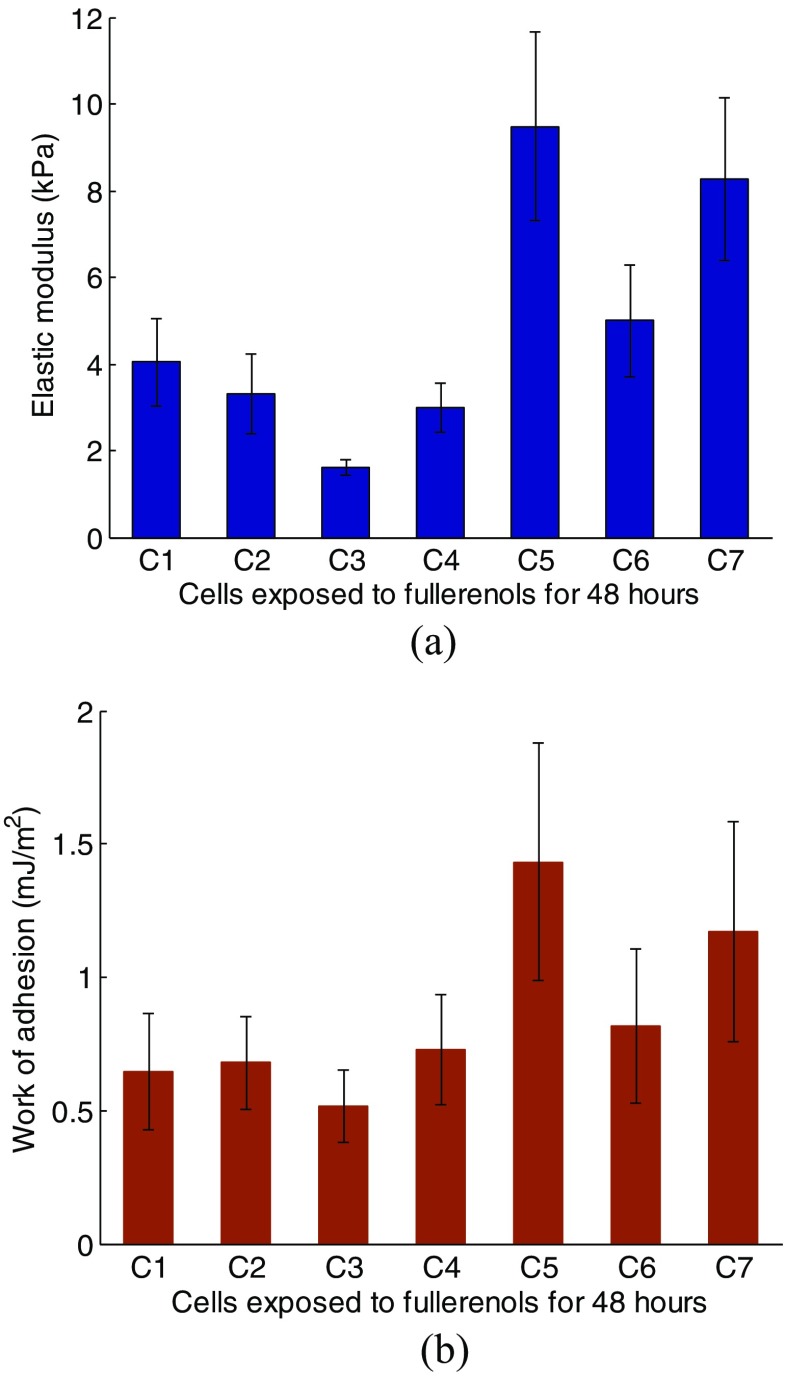
Histograms showing the determined (**a**) Young’s modulus and (**b**) work of adhesion for each cell *C*

In this study we explore two ways of fitting the unloading part of *F-d* curve as detailed below. The first way is fitting from beginning of retraction to the place where the indentation force exhibits severe discontinuities as shown by the big plot in Fig. 7, termed as “global fitting”. The second method is fitting the fraction of *F-d* curve from the point where indentation force decreases to null to where force is significantly discontinuous (this “swale” area corresponds to low indentation depth), as shown by the zoom box area in Fig. 8, termed as “local fitting”. The *R-square* value in the zoom box corresponds to the fit goodness when the extracted parameters produced by “global fitting” are used to describe the “swale” area. It is suggested that when the *R-square* value in the zoom box is around 0.5 or even higher, there are no significant difference between the fitting results by the two approaches as illustrated by Fig. 8a-c. However, when this value is fairly small, significant variation of extracted parameters is observed between the two methods as shown by Fig. 8d. Therefore, it can be concluded that in this “swale” area, adhesion force plays a dominant role for fitting result and has a considerable effect on the extracted parameters.

**Fig. 9:**
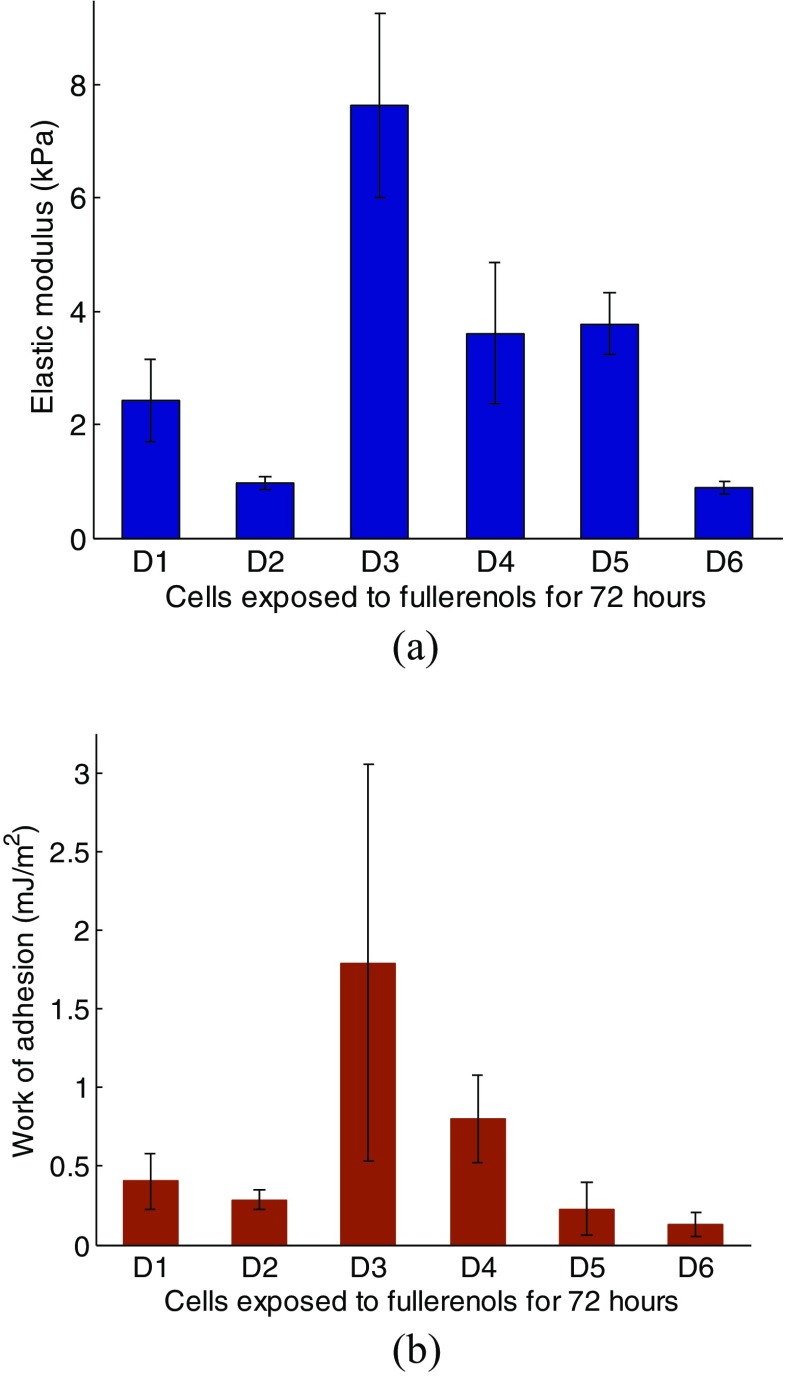
Results of the determined (**a**) Young’s modulus and (**b**) work of adhesion for each cell *D*

**Fig. 10:**
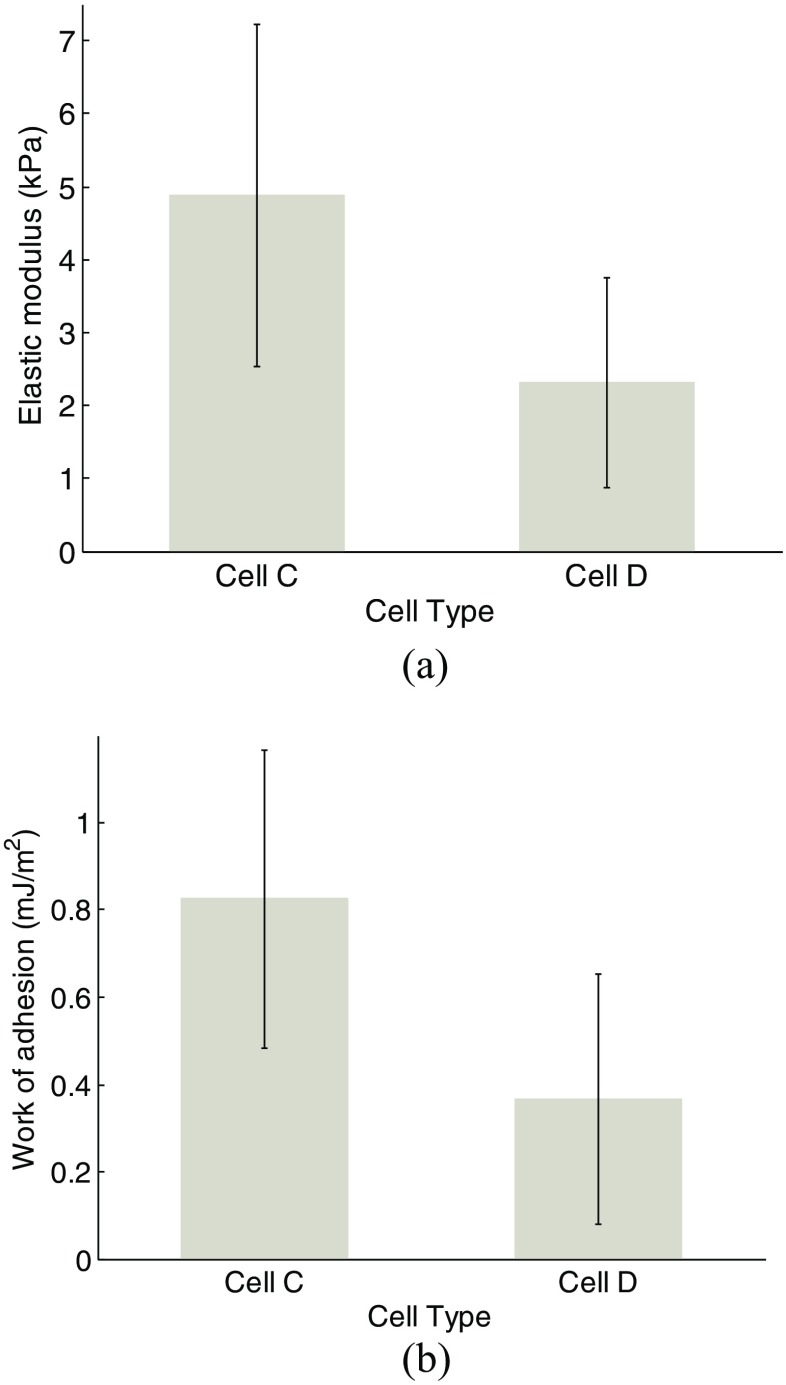
The comparison of determined (**a**) Young’s modulus and (**b**) work of adhesion between cells *C* and *D*

Tables 3, 4, 5, and 6 list the extracted Young’s modulus and work of adhesion corresponding to one cell group (cells *C* or *D*) by the “global fitting” method. It indicates that these two parameters do not exhibit significant differences within one cell in adhesion circumstance. Likewise, we performed statistical analysis on *F-d* curve corresponding to each cell, and the extracted Young’s modulus and work of adhesion, as shown in Figs. 8 and 9. The average value of the two parameters varies from one cell to another, and cells with larger Young’s modulus exhibits larger work of adhesion approximately. For cell *C*, the Young’s modulus and work of adhesion mostly range between 3 to 5 kPa and 0.5 to 0.8 mJ/m^2^, respectively. For cell *D*, the Young’s modulus and work of adhesion mostly range between 1 to 4 kPa and 0.1 to 0.4 mJ/m^2^, respectively. Figure 10 shows the statistics results (average value) taking all cells into consideration. For cell *C* Young’s modulus and work of adhesion have an average of 4.88kPa and 0.825 mJ/m^2^ respectively, while for cell *D* Young’s modulus and work of adhesion have an average of 2.32kPa and 0.365 mJ/m^2^ respectively. The determined value of work of adhesion in our procedure can almost coincide with the value in a former study [19] in order of magnitude which in turn justifies this procedure. The difference between the heights of histograms suggests that both cell stiffness and adhesion effect is decreased by fullerenol treatment during the last 24 h.

**Table 3:** Young’s modulus from cell *C2* (Unit: kPa)

Time \ spot	1st	2nd	3rd	4th	Avg (Std)
1	2.99	3.12	2.99	3.64	3.19(±0.31)
2	3.74	4.05	4.38	-	4.06(±0.32)
3	2.8	3.74	3.53	-	3.36(±0.49)
4	3.64	4.99	4.73	-	4.45(±0.72)
5	2.2	2.33	2.99	2.88	2.6(±0.39)

**Table 4:** Work of adhesion from cell *C2* (Unit: mJ/m^2^)

Time \ spot	1st	2nd	3rd	4th	Avg (Std)
1	0.789	0.592	1	0.48	0.715(±0.229)
2	1	0.758	0.572	-	0.777(±0.215)
3	0.744	0.699	0.796	-	0.746(±0.049)
4	0.733	0.789	0.655	-	0.726(±0.067)
5	0.558	0.423	0.482	0.556	0.505(±0.065)

**Table 5:** Young’s modulus from cell *D2* (Unit: kPa)

Time\ spot	1st	2nd	3rd	4th	Avg (Std)
1	1.15	1.11	0.925	0.84	1.01(±0.15)
2	1.01	0.92	1.09	1.16	1.05(±0.10)
3	1.08	0.96	1.05	0.97	1.02(±0.06)
4	0.93	0.97	0.83	0.98	0.93(±0.07)
5	0.83	0.8	0.84	0.88	0.84(±0.03)

**Table 6:** Work of adhesion from cell *D2* (Unit: mJ/m^2^)

Time\ spot	1st	2nd	*3rd*	4th	Avg (Std)
1	0.234	0.252	0.339	0.275	0.275(±0.046)
2	0.305	0.227	0.358	0.244	0.284(±0.06)
3	0.338	0.319	0.345	0.267	0.317(±0.035)
4	0.199	0.361	0.308	0.148	0.254(±0.098)
5	0.294	0.362	0.273	0.308	0.309(±0.038)

**Fig. 11:**
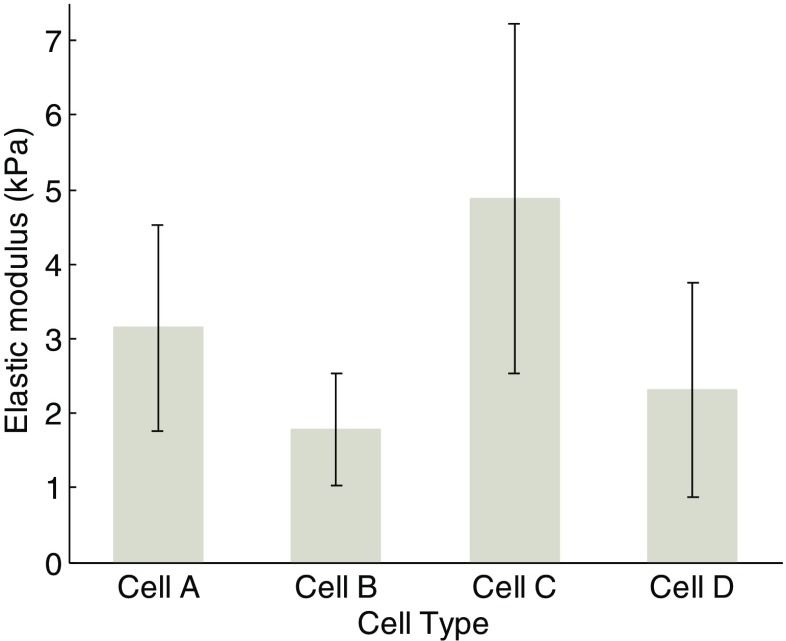
Results of Young’s moduli of the four group cells by using JKR model

**Fig. 12:**
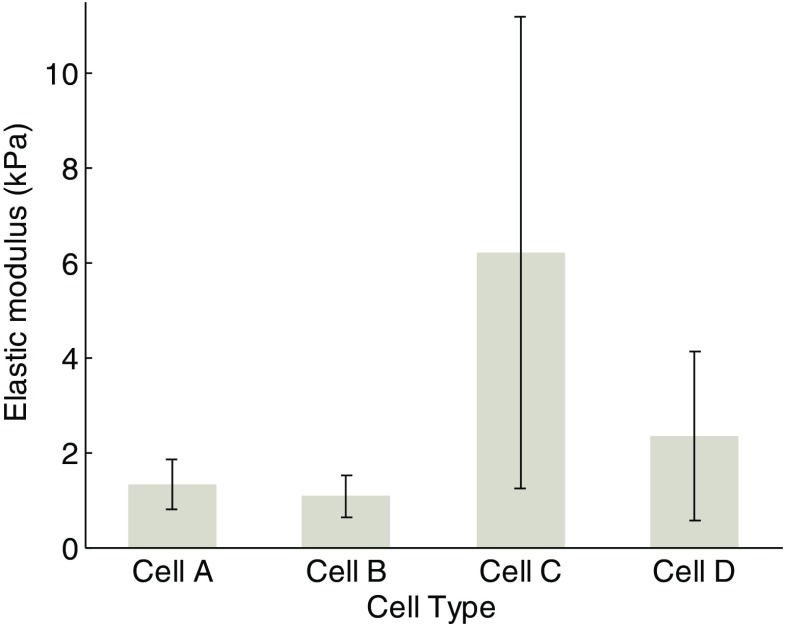
Results of Young’s moduli of the four group cells, by using Hertzian contact model to fit the loading parts of *F-d* curves

Figure 11 is the combination of Figs. 5 and 10a. The effect of duration of fullerenol treatment on extracted modulus seems different from that reported by a former study [6]. In general, the Young’s modulus is derived from the loading curve of the f-d curves by Herzian contact model. In order to make a comparison, we have tried to apply the Hertzian contact model to fit the loading part of the *F-d* curves of the four groups of cells. The results (average ± std) are plotted in Fig. 12. It can be seen that the pattern of the calculated Young’s modulus values of the four group cells are almost the same as that in Fig. 11. Therefore, the difference is more likely due to the variation of different batch of cells and the fullerenols treatment.

## Conclusion

In this study, AFM nanoindentation was employed to investigate the mechanical properties of human hepatocellular carcinoma cells treated with fullerenol for 24, 48 and 72 h. For each cell, 8–10 nanoindentations were carried out (3–5 indents on the same spot and 5 indents at different spots). The results show that the measured elastic modulus value varies mainly with the type of cells. The shift of *F-d* curves from the same cell is much likely due to height (thickness) of the cell. The controlled cells (cells A) and treated cells B showed non-adhesive *F-d* curves, therefore, Hertz contact model was applied. On the other hand, the cells treated with fullerenol for 48 and 72 h showed significant adhesion and thus JKR model was applied to fit the corresponding retraction region of the *F-d* curves. The results show that Hertz and *JKR* contact models can both fit very well the experimental data in each case. In non-adhesion case of cells B, fitting by Hertz model indicated 24 h treatment of fullerenol may make the treated cells more compliant. With the presence of adhesion force in cells C and D, “global” fitting by the JKR model suggested both stiffness and adhesion of the treated cells were decreased by a large margin during the last 24 h treatment of fullerenol. Therefore, the results suggest that the experimental study of cell-tip adhesion may also provide some insights into potential cancer progression in addition to cell stiffness. The derived mechanical properties of elastic modulus and work of adhesion could be used as an effective bio-index to evaluate the effects of fullerenol or other anticancer agents on cancer cells and thus to provide insight into cancer progression in the treatment.
